# Role of retinoic acid receptors in squamous-cell carcinoma in human esophagus

**DOI:** 10.1186/1477-3163-4-20

**Published:** 2005-11-08

**Authors:** I Bergheim, E Wolfgarten, E Bollschweiler, AH Hölscher, Ch Bode, A Parlesak

**Affiliations:** 1 Department of Physiology of Nutrition, Hohenheim University (140e), Garbenstrasse 28, 70599 Stuttgart, Germany; 2 Department of Visceral and Vascular Surgery, University of Cologne, Joseph-Stelzmann-Strasse 9, 50931, Cologne, Germany

## Abstract

**Background:**

Worldwide, cancer in the esophagus ranks among the 10 most common cancers. Alterations of retinoic acid receptors (e.g. RARα, β, γ, and RXRα, β, γ) expression is considered to play an important role in development of squamous-cell carcinoma (SCC), which is the most common esophageal cancer. Alcohol consumption and smoking, which can alter retinoic acid receptor levels, have been identified as key risk factors in the development of carcinoma in the aero-digestive tract. Therefore, the aim of the present study was to evaluate protein levels of retinoic acid receptors (i.e. RARα, β, γ, and RXRβ) in esophageal SCC and surrounding normal tissue of patients with untreated SCC and controls.

**Methods:**

All study participants completed a questionnaire concerning smoking and alcohol drinking habits as well as anthropometrical parameters. Protein levels of RARα, β, γ, and RXRβ were determined by Western Blot in normal esophageal tissue and tissue obtained from SCC of 21 patients with newly diagnosed esophageal SCC and normal esophageal tissue of 10 controls.

**Results:**

Protein levels of RARγ were significantly lower by ~68% in SCC compared to normal surrounding tissue in patients with SCC that smoked and/or consumed elevated amounts of alcohol. Furthermore, RARα protein levels were significantly lower (~- 45%) in SCC in comparison to normal esophageal mucosa in patients with elevated alcohol intake. When comparing protein levels of retinoic acid receptors between normal tissue of patients with SCC and controls, RARγ protein levels were found to be significantly higher (~2.7-fold) in normal esophageal tissue of SCC patients than in esophageal tissue obtained from controls. No differences were found for RARα, β, and RXRβ protein levels between normal esophageal tissue of patients and that of controls.

**Conclusion:**

In conclusion, results of the present study suggest that alterations of retinoic acid receptors protein may contribute in the development of SCC in esophagus and that in some patients life style (e.g. smoking and alcohol consumption) may be a critical component in the alteration of retinoic acid receptor levels in esophagus.

## Background

Worldwide esophageal cancer ranks among the ten most common cancers [1] and the overall 5-year survival rate for patients diagnosed with esophageal cancer is poor (e.g. 3%–10%) [2]. Although, there is a rising entity of adenocarcinoma [3], the majority of carcinoma of the esophagus are squamous-cell carcinoma (SCC) [1]. Multiple epidemiological studies indicate that alcohol and tobacco may be important risk factors for the development of carcinoma in the esophagus [1,4]. In a case-control study, Valsecchi et al. [5] showed that people who smoked 40 or more years had almost 6-times the risk of esophageal cancer development compared to non-smokers. Those with a history of alcohol consumption of more than two beers per day had 3-times the risk in comparison to abstainers. Furthermore, the combination of both habits was found to have a synergistic effect, increasing the relative risk for developing cancer in the aero-digestive tract up to 17.3-times over that of the non-smokers, non-drinkers [6]. However, despite intense research [7], molecular pathomechanisms involved in the development of esophageal SCC are not fully understood so far contributing to the lack of successful pharmacological therapies.

Alterations of retinoid metabolism and retinoic acid receptor (e.g. RARα, β, γ, and RXRα, β, γ)-mediated gene transcription have been proposed to play an important role in the pathogenesis of SCC [8-10]. It is well established that RARs and RXRs are ligand-dependent transcription factors with distinct expression patterns in early and adult stages of development. Therefore, down-regulation (e.g. loss or low expression of the specific nuclear receptor) or "functional" down-regulation (e.g. lack of ligands) of both retinoic acid receptor families could interfere with the retinoid signal transduction and, over time, might result in enhanced cell proliferation and malignant transformation. An association between altered expression of nuclear retinoic acid receptors and the malignant transformation of human cells has been demonstrated in several studies [11-14]. Furthermore, a reduced expression of RARγ found in head and neck SCC cell lines [15] seems to depend on a sufficient Vitamin A supplementation [16].

Since little is known about the abundance of RARs and RXRs protein in esophageal SCC in humans, the major focus of the present study was to determine protein levels of RARα, β, γ, and RXRβ in normal, macroscopically unaffected esophageal tissue and SCC of patients with untreated SCC and controls. In addition, alcohol intake and smoking habits of patients and controls were evaluated and related to retinoic acid receptor levels.

## Materials and methods

### Subjects and tissue specimens

The study was approved by the Ethics Committee of the Medical Clinic of the University of Cologne, Germany. Informed consent was obtained from all subjects included in the study. A total of 31 subjects, all of whom were undergoing endoscopies for medical reasons, were included in the study. Twenty-one patients had untreated SCC of the esophagus, and ten SCC-free subjects served as controls. Histopathological analysis of SCC was performed by an experienced physician. Tumor staging was based on a graded rating system and varied from G1 (well differentiated) to G3 (poorly differentiated). In controls, the presence or absence of SCC was confirmed macroscopically. All study participants completed a questionnaire concerning smoking and alcohol drinking habits as well as anthropometrical parameters. Using standard pinch forceps, two biopsies were obtained from macroscopically normal esophageal tissue and SCC of subjects with carcinoma and from the mucosa of healthy controls. Biopsies were placed immediately in liquid nitrogen and stored at -80°C until analysis.

### Immunoblot Analysis

Total protein was isolated using Trizol reagent (Invitrogen, Carlsbad, CA, USA). Using SDS-polyacrylamide gel electrophoresis, 20–30 μg of total protein were separated in a 9% polyacrylamide gel (PAGE). Protein was transferred to a nitrocellulose membrane (Amersham/ Pharmacia, Freiburg, Germany). Membranes were blocked in Tris-buffered saline (TBS) with 5% non-fat milk powder with Tween 20 (TBST, 0.01% v/v Tween 20), rinsed in TBST, and probed with primary antibodies dissolved in TBS followed by an incubation with the secondary antibody. Primary antibodies against RARα, β, γ, and RXRβ were obtained from Alexis (Dianova, Hamburg, Germany), the secondary peroxidase-conjugated antibodies were purchased from Boehringer (Mannheim, Germany). Bands were visualized by enhanced chemiluminescence using SuperSignal^® ^West Dura (Pierce, KTM, Bad Godesberg, Germany). Blots were photographed (Camera LAS 1000, Fuji, USA) and immunoquantitation was accomplished by densitometric analysis using the software AIDA (Raytest, Isotopenmessgeraete, Straubenhardt, Germany). To achieve standardization and comparability of blots, total protein derived from human liver was loaded on each gel at three concentrations (50 μg, 25 μg, and 12.5 μg) and blottet with the samples. To ensure equal loading, all blots were stained with Ponceau red; haptene signals were normalized to β-actin using a commercially available antibody (Sigma Chemical Co., Munich, Germany).

### Statistical Analysis

Results are presented as mean ± standard error of the mean (SEM), unless otherwise stated. Wilcoxon *t *test was used to test for significance of differences in expression levels measured in both normal and malignant tissue of patients with SCC. Fisher's exact test was used for comparison of lifestyle data. Statistical comparison of values originating from separate groups was performed with the Mann-Whitney *U*-test. *P *< 0.05 was considered to represent a significant difference.

## Results

Subject age ranged from 38–71 years and most were of normal weight (Table 1). Smoking habits did not differ between patients and controls. However, significantly more patients with SCC reported to consume "elevated" amounts of alcohol (more than 30 g of alcohol/d or more 4-times a week more than 1 drink) in comparison to controls (Table 1). Tumor grading ranged form G1 to G3 with the majority being G2 (Table 2).

**Table 1:** Characteristics of patients with untreated esophageal SCC and controls.

**Parameter**	**Patients**	**Controls**	**p-values**
n	21	10	
Age	56.7 ± 1.9	51.1 ± 3.5	0.118
Sex (female/male)	7/14	5/5	0.425
BMI	23.8 ± 1.4	25.1 ± 0.6	0.370

Cigarette usage (yes/no)	10/11	3/7	0.242
Alcohol consumption^*)^			
none	4^**)^	2	**0.012**
moderate	8	8	
elevated	9	0	

All data expressed as mean ± SEM. (BMI = body mass index, p < 0.05 statistically significant) ^*) ^none = study subject reported to never drink alcohol; moderate = study subject reported to drink alcohol sometimes (not more than once a week); elevated = study subject reported to consume alcohol more than 4-times a week (more than one drink) or more than 30 g ethanol/d on the average ^**) ^two patients had been former heavy alcohol abusers but reported to have given up alcohol consumption several years ago

**Table 2:** Tumor staging of patients with SCC.

**Tumor stage**	**n**
**G1**	2
**G1-2**	2
**G2**	8
**G2-3**	4
**G3**	5

G1 = well differentiated

G2 = moderately differentiated

G3 = poorly differentiated

### Retinoic acid receptor protein levels in patients with untreated esophageal SCC

**Figure 1:**
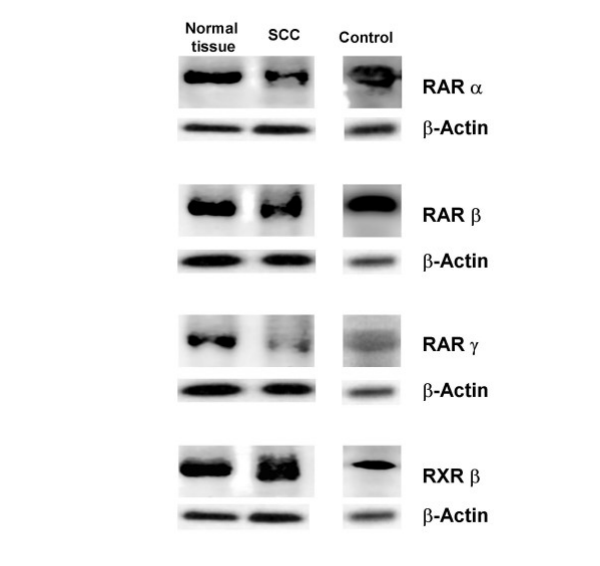
**RARα, β, γ, and RXRβ protein in human esophagus. **Representative Western blot of RARα, β, γ, and RXRβ protein in normal esophagus of controls and normal, unaffected esophageal tissue of patients with untreated esophageal SCC and SCC. Protein levels of β-actin were determined as loading control.

**Figure 2:**
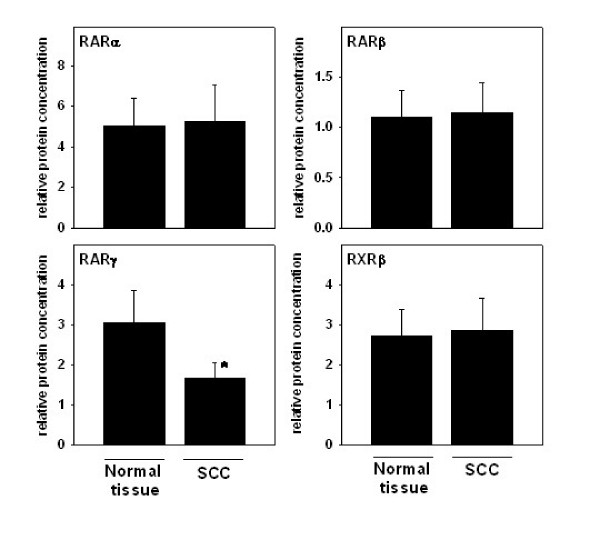
**Protein levels of RARα, β, γ, and RXRβ in patients with esophageal SCC. **Quantitative analysis of Western blots performed in normal tissue of patients with SCC and tissue obtained form SCC. Protein levels of β-actin were determined as loading control. Data are means ± SEM. *p < 0.05

Figures 1 and 2 summarize the results of protein measurements performed in biopsy specimens obtained from SCC and neighboring normal esophageal tissue of patients with untreated esophageal SCC. Western Blot analyses demonstrated that RARα, β, γ, and RXRβ were present as a major immunoreactive band representing the 55 kDa monomer in all tissue samples tested (see Figure 1). Protein levels of RARγ were found to be significantly lower in specimens obtained from SCC in comparison to surrounding normal tissue. Specifically, RARγ protein levels were ~47% lower in SCC when compared with those of normal, unaffected esophageal tissue (p = 0.040). In contrast, no significant differences were found when comparing protein levels of RARα, RARβ, and RXRβ between macroscopically normal, unaffected esophageal tissue and SCC.

**Figure 3:**
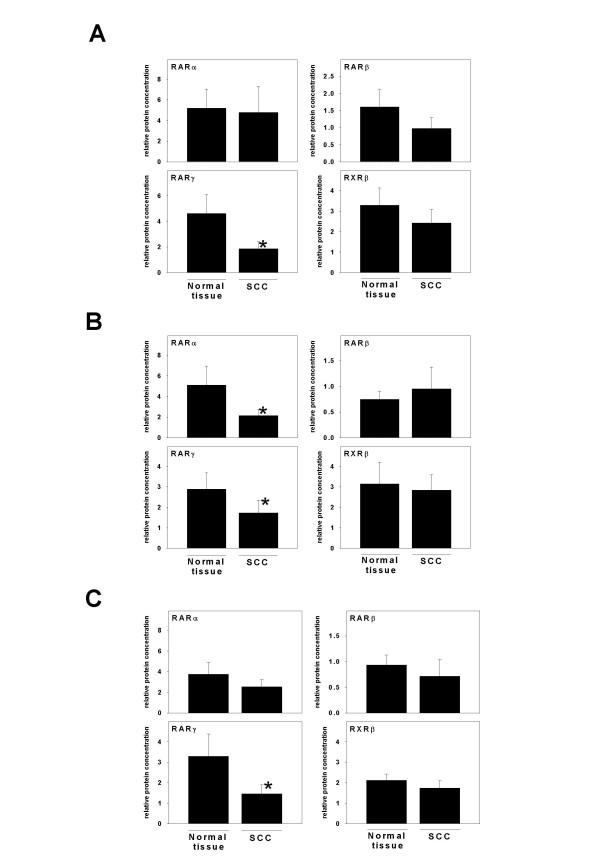
**Protein levels of RARα, β, γ, and RXRβ in smoking and alcohol consuming patients with esophageal SCC. **Quantitative analysis of RARα, β, γ, and RXRβ protein levels in normal unaffected esophageal tissue und SCC of (A) smoking SCC patients, (B) patients with "elevated" alcohol consumption, and (C) smoking SCC patients with "elevated" alcohol intake. Protein levels of β-actin were determined as loading control. Data are means ± SEM. *p < 0.05 (Normal tissue = macroscopically unaffected tissue, SCC = squamous-cell carcinoma)

To investigate whether smoking and alcohol consumption influences protein levels of esophageal retinoic acid receptors of patients with SCC were grouped by smoking habits (e.g. non-smoker or smoker) regardless of their alcohol consumption. In smokers, RARγ protein levels were found to be significantly lower (~68%, p = 0.010) in SCC in comparison to unaffected esophageal tissue in smoking patients (n = 10). Interestingly, a similar difference was not found in patients who were non-smokers (n = 11, data not shown). Similar, patients were than sub-grouped by alcohol intake regardless of their smoking habits into patients who (a) reported to consume no alcohol (= "none"), (b) who reported to be "moderate" alcohol consumers not exceeding alcohol intake more than once a week, or (c) who stated to consume alcohol more than 4-times a week or more than 30 g raw ethanol/ d (see also Table 2). Since only 4 patients reported to be abstainers and since two patients in this group stated to have given up heavy alcohol consumption several years ago, this group was excluded from the statistical analysis. Results are summarized in Figure 3. When comparing protein levels of retinoic acid receptors between SCC and unaffected tissue obtained from patients who reported to consume "elevated" amounts of alcohol (n = 9), RARγ protein levels were again found to be significantly lower in SCC in comparison to neighboring normal tissue (~40%, p = 0.038). Furthermore, in this subgroup of patients, in biopsies obtained from SCC protein levels of RARα were ~55% lower in comparison to normal tissue (p = 0.049). Again, similar to non-smokers, in patients who were moderate consumers of alcohol (n = 8), neither protein levels of RARα nor RARγ were found to differ between tissue obtained from SCC and normal esophageal tissue (data not shown). Next, receptor protein levels were compared between SCC and normal tissue of patients who consumed elevated amounts of alcohol and smoked (n = 6). Interestingly, only levels of RARγ were found to be significantly lower by ~68% in esophageal SCC in comparison to normal esophageal tissue (p = 0.046). As to be expected, in patients, who had a "moderate" alcohol consumption and were non-smokers, no differences were found in protein levels of RARs or RXRβ.

### Retinoic acid receptor protein levels in normal tissue of patients with esophageal SCC and controls

**Figure 4:**
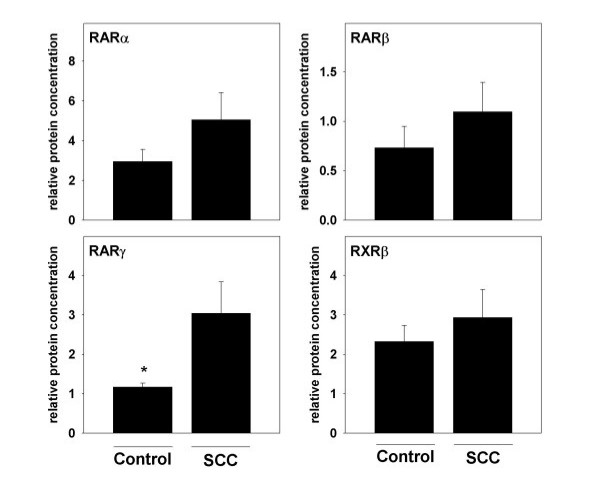
**RARα, β, γ, and RXRβ protein levels in normal esophageal tissue of patients with SCC and controls. **Quantitative analysis of RARα, β, γ, and RXRβ protein levels as determined by Western blot. Protein levels of β-actin were determined as loading control. Data are means ± SEM. *p < 0.05

Since it has been suggested by the results of others (19) that basal levels of retinoic acid receptor mRNA expression in normal unaffected tissue might differ between patients with SCC and controls, protein levels of RARα, β, γ, and RXRβ were determined in esophageal tissue obtained form controls and compared with protein levels measured in normal esophageal tissue obtained from patients with esophageal SCC. Representative Western blots are depicted in Figure 1 A and quantitative analysis of blots is summarized in Figure 4. Analysis revealed a significantly higher protein concentration of RARγ in normal unaffected tissue of patients with esophageal SCC in comparison to controls. Specifically, protein levels were found to be ~2.7-fold higher in normal tissue of patients when compared with controls (p = 0.048). No significant differences were found when comparing protein levels of RARα, γ, and RXRβ measured in normal, macroscopically unaffected tissue specimens of patients and controls.

## Discussion

### Alterations of RARγ and RARα protein levels in patients with esophageal SCC are depending on life style

In the present study, the hypothesis was tested that altered protein levels of retinoic acid receptors might be involved in the development of esophageal SCC in humans. Indeed, protein levels of RARγ were found to be significantly lower in SCC relative to normal tissue in patients who smoked and consumed alcohol. A similar difference was found for RARα in SCC patients who reported to consume "elevated" amounts of alcohol. However, diminished levels of RARγ and RARα were only found in these sub-populations of patients with SCC in the esophagus, suggesting that underlying mechanism leading to the development of SCC may vary depending on life style of patients. Several groups have previously determined mRNA expression of RARs and RXRs in normal esophageal tissue and SCC. Zhang et al. [17] and Qiu et al. [18], who measured mRNA levels of RARs and RXRs found a striking reduction of the expression of RARβ but not of RARγ in malignant esophageal tissue in comparison to normal tissue. Furthermore, in *in situ *studies a reduced mRNA expression of RARα was found in esophageal SCC compared with normal tissue [11]. However, in these studies, no information was given on life style parameters such as smoking and alcohol intake and patients were not sub-grouped accordingly and only mRNA expression levels of RARs and RXRs were evaluated.

Due to the retrospective character of the present study it can only be speculated whether the association of cigarette smoking and reduced protein levels of RARγ in SCC is a cause – and effect relationship or not. However, the action of tobacco carcinogens are believed to be mediated through covalent binding to DNA, RNA, or protein, forming DNA, RNA, and protein adducts [19]. Furthermore, results of *in vitro *and *in vivo *studies [20] suggest that tobacco carcinogens (e.g. benzo(a)pyrene diol epoxide) might lead to DNA methylation of RAR genes subsequently resulting in an inhibition of the expression of RARs rather than mediating their action through direct mutations.

Several mechanism have been proposed to explain how alcohol ingestion may interfere with retinoid metabolism. First, it has been shown that alcohol may act as a competitive inhibitor of oxidation of retinal to retinoic acid in several organs (e.g. colon and esophagus) [21-23]. Secondly, Liu et al. [24] reported an enhanced catabolism of retinoids into polar metabolites in the liver of ethanol-fed rats compared to pair-fed animals. This was found to be inhibited *in vitro *and *in vivo *by chlormethiazol, an inhibitor of cytochrome P4502E1 (CYP2E1). A significant induction of CYP2E1 was found after one week of chronic alcohol ingestion in human subjects [25]. Therefore, the induction of CYP2E1 (and/or other cytochrome P450 isoforms) activity during chronic alcohol intake could add to a reduced bioavailability of retinoic acid subsequently resulting in changes in the expression of RARs and RXRs. Thirdly, it has been shown that levels of cellular retinoic acid binding protein (CRABP) can be reduced in esophageal carcinoma (e.g. adenocarcinoma and SCC) [26]. This, in turn, may lead to alterations of receptor expression and turnover. However, whether these mechanisms play a role in the present study remains to be determined.

### RARγ protein levels are elevated in normal esophageal mucosa of patients with SCC

Untill now, information on protein levels of retinoic acid receptors in normal unaffected esophageal tissue has been limited. Recently, in a study determining the role of RXR mRNA in Barett's esophagus mRNA levels of RXRβ were found to be higher in non-malignant tissue in patients with adenoma than in healthy controls [27]. In the present study, protein levels of RARγ were found to be significantly higher in normal esophageal tissue of patients with esophageal SCC in comparison to controls. It may be that the differences between the results of others [27] and the present study are due to differences of patients (e.g. patients with SCC vs. patients with Barrett esophagus) or differences of detection sensitivity (mRNA expression vs. protein).

## Conclusion

The results of the present study provide further evidence that a diminished abundance of retinoid acid receptors in esophagus is associated with the development of esophageal SCC in humans. Furthermore, in some patients life style (e.g. smoking and alcohol consumption) may contribute to the alteration of retinoic acid receptor levels in esophagus. Further studies will be required to elucidate this interaction of alcohol consumption, smoking, and retinoic acid receptor signaling with the development of SCC in the esophagus.

## Competing interests

The author(s) declare that they have no competing interest.

## Authors' contributions

IB has made substantial contributions to the biochemical analysis and the drafting of article. EW has substantially contributed to the acquisition of data, and conception as well as design of the study. EB, AHH, and CB have made substantial contributions to conception and design as well as the interpretation of data. AP has been involved in the design, the drafting of the article, and revised it critically for important intellectual content. All authors have given final approval of the version to be published.
